# Synthesis, Characterization and Magnetic Hyperthermia of Monodispersed Cobalt Ferrite Nanoparticles for Cancer Therapeutics

**DOI:** 10.3390/molecules25194428

**Published:** 2020-09-27

**Authors:** Mauricio A. Medina, Goldie Oza, A. Ángeles-Pascual, Marlene González M., R. Antaño-López, A. Vera, L. Leija, Edilso Reguera, L. G. Arriaga, José Manuel Hernández Hernández, José Tapia Ramírez

**Affiliations:** 1 Program on Nanoscience and Nanotechnology, CINVESTAV-IPN, Avenida IPN 2508, Gustavo A. Madero, San Pedro Zacatenco, Mexico City 07360, Mexico; 2 Centro de Investigación y Desarrollo Tecnológico en Electroquímica (CIDETEQ), Parque Tecnológico Querétaro s/n, Sanfandila, Pedro Escobedo, Querétaro C.P. 76703, Mexico; rantano@cideteq.mx (R.A.-L.); larriaga@cideteq.mx (L.G.A.); 3 Laboratorio Avanzado de Nanoscopía Electrónica-LANE, Centro de Investigación y de Estudios CINVESTAV-IPN, Avenida IPN 2508, Gustavo A. Madero, San Pedro Zacatenco, México City 07360, Mexico; aangelesp@cinvestav.mx; 4 CONACyT-Instituto Politécnico Nacional, Centro de Investigación en Ciencia Aplicada y tecnología Avanzada, U. Legaria, Ciudad de México 11500, Mexico; maglerne@gmail.com (M.G.M.); edilso.reguera@gmail.com (E.R.); 5 Bioelectronics Section, Department of Electrical Engineering, CINVESTAV-IPN, Avenida IPN 2508, Gustavo A. Madero, San Pedro Zacatenco, México City 07360, Mexico; arvera@cinvestav.mx (A.V.); lleija@cinvestav.mx (L.L.); 6 Department of Cellular Biology, CINVESTAV-IPN, Avenida IPN 6508, San Pedro Zacatenco, Mexico D.F. 07360, Mexico; manolo@cell.cinvestav.mx; 7 Department of Genetics and Molecular Biology, CINVESTAV-IPN, Avenida IPN 2508, Gustavo A. Madero, San Pedro Zacatenco, México City 07360, Mexico

**Keywords:** nanomedicine, thermal decomposition, TNBC, hyperthermia, CoFe_2_O_4_

## Abstract

Magnetic nanoparticles such as cobalt ferrite are investigated under clinical hyperthermia conditions for the treatment of cancer. Cobalt ferrite nanoparticles (CFNPs) synthesized by the thermal decomposition method, using nonionic surfactant Triton-X100, possess hydrophilic polyethylene oxide chains acting as reducing agents for the cobalt and iron precursors. The monodispersed nanoparticles were of 10 nm size, as confirmed by high-resolution transmission electron microscopy (HR-TEM). The X-ray diffraction patterns of CFNPs prove the existence of cubic spinel cobalt ferrites. Cs-corrected scanning transmission electron microscopy–high-angle annular dark-field imaging (STEM–HAADF) of CFNPs confirmed their multi-twinned crystallinity due to the presence of atomic columns and defects in the nanostructure. Magnetic measurements proved that the CFNPs possess reduced remnant magnetization (M_R_/M_S_) (0.86), which justifies cubic anisotropy in the system. Microwave-based hyperthermia studies performed at 2.45 GHz under clinical conditions in physiological saline increased the temperature of the CFNP samples due to the transformation of radiation energy to heat. The specific absorption rate of CFNPs in physiological saline was 68.28 W/g. Furthermore, when triple-negative breast cancer cells (TNBC) in the presence of increasing CFNP concentration (5 mg/mL to 40 mg/mL) were exposed to microwaves, the cell cytotoxicity was enhanced compared to CFNPs alone.

## 1. Introduction

Magnetic nanoparticles (MNPs) are attracting much scientific attention due to their application in the fields of theranostic imaging, cell tracking, MRI, hyperthermia, drug delivery, giant magnetoresistance-based biosensors, and the separation of biomolecules such as DNA and proteins [1,2,3,4]. Uniquely, MNPs possess inverse spinel structures in the form of MFe_2_O_4_, where M corresponds to Co^+2^, Fe^+2^, Ni^+2^, Mn^+2^, etc., and inhabits either tetrahedral or octahedral interstitial sites. There is a need to accurately detect these M^+2^ chemical species, because they can enhance the magnetic properties of the particles by increasing the magnetic permeability or electrical resistivity [5,6,7,8,9]. Cobalt ferrite is a well-known ferrite that shows room temperature coercivity as well as very high magneto-crystalline anisotropy; thus, it is an important asset for cancer chemotherapy as well as hyperthermia treatment [10,11]. MNPs exhibit relaxation, which can occur via two mechanisms: Brownian (relaxation time in which the magnetic dipole rotates in the form of a rigid body and is completely locked inside the particle) or Neel relaxation (switching of the magnetic dipole between two easy axes with respect to time). MNPs under an alternating current (AC) magnetic field exhibit heating effects due to the losses when there is a reversal of magnetization taking place in the magnetic nanoparticles [12,13,14].

According to the National Cancer Institute (NCI), localized hyperthermia can be induced using microwaves, radiofrequency, ultrasound and even light. The disadvantage of traditional hyperthermia techniques using radiofrequency and microwaves are that they are invasive and lead to serious side effects. Another modality of hyperthermia is light that causes photothermal treatment using infra-red radiation, generating heat and inducing the necrosis or apoptosis of cancer cells. Again, the disadvantage of light is the lack of depth in its penetration into the tissues, thus proving to be futile for deep tumor therapy. Gold nanoparticles, especially gold nanospheres [15], gold nanostars [16], gold nanoshells [17] and gold nanorods [18], single-walled carbon nanotubes [19], multiwalled carbon nanotubes [20], and polymers such as polyaniline [21] can be used for photothermal cancer therapy as they can be injected and reach the local tumor area in a targeted manner and can be stimulated via near infra-red laser light to generate heat. There are magnetic nanoparticles such as iron oxide [22,23], doped iron oxide [24,25] and superparamagnetic iron oxide nanoparticles [26] that can be stimulated via alternating magnetic fields or microwaves to generate heat. Such magnetic nanoparticles can generate hyperthermia and increase the surrounding temperature to 42–45 °C. The mechanism of heat dissipation using MNPs involves a delay in the relaxation of the magnetic moments, either due to rotation within the particle (Néel) or due to rotation of the particle itself (Brownian) when an AC magnetic field is applied, in which the magnetic field reversal time is shorter than the magnetic moment relaxation of the nanoparticles [27,28]. In this article, we are exploiting cobalt ferrite nanoparticles for its cytotoxicity against the triple-negative breast cancer cell line (MDA-MB-231) in the presence of microwaves (2.45 GHz) since the exposure time for cell mortality is lesser compared to other hyperthermia techniques and CFNPs can be more precise in terms of killing cancer cells if properly targeted. Cobalt enhances the magnetic properties of the magnetic ferrites, and thus also increases the output of hyperthermia considerably [29,30]. Cobalt ferrites have a slower magnetic moment relaxation when compared to magnetite. Moreover, when such ferrites are altered by different biocompatible surface modifiers such as pullulan [3], chitosan [4,31,32,33], polyethylene glycol [2,4,34,35,36], dextran [36], etc., then their biocompatibility is further enhanced.

The specific absorption rate (SAR) is the most important characteristic of any magnetic particle and can be defined as the rate of the electromagnetic energy absorption by a unit mass of biological material, with units of watts (W) per kilogram (kg). Moreover, the SAR is also proportional to the rate of the temperature rise (ΔT/Δt) due to energy absorption, when measurements are carried out in a short time and therefore the thermal conduction effects are minimal. Hence:

SAR = [CeV] × (dT/dt)(1)

This relation is obtained from the Pennes bioheat equation. Using Equation (1), SAR is derived from P, which is the power absorbed by the material, and Ce, the specific heat capacity of the material, V is the volume of the sample, DT/dt is the change in temperature with respect to time [29,30].

In the present article, cobalt ferrite was first synthesized by a thermal decomposition (TD) route, using Triton X-100 as a surfactant to form the nanoparticles. These nanoparticles (NPs) possess good crystalline anisotropy, excellent saturation magnetization, mechanical hardness, high Curie temperature, high magnetic hyperthermia, and good biocompatibility. The cobalt ferrite nanoparticles (CFNPs) were characterized optically, magnetically, and structurally, and then hyperthermia studies were performed in vitro, in the presence and absence of triple negative breast cancer (TNBC) cells (MDA-MB-231), as well as fibroblast cells (MRC-5).

## 2. Results and Discussion

### 2.1. Mechanism of CFNP Synthesis

In this study, thermal decomposition was used as the synthesis method for homogeneous CFNP formation. There are many reaction parameters that play an important role in the successful synthesis and stabilization of the NPs such as the length of the reflux time, temperature, type of surfactant, type of solvent, the gas in which the reaction is performed, and concentration of the precursors. The size and shape of the nanoparticles are particularly determined by the reflux temperature, time, the type of surfactant and solvent [37].

Cobalt acetylacetonate and iron acetylacetonate react with the nonionic surfactant Triton-X100, which possesses hydrophilic polyethylene oxide chains that act as both reducing and capping agents. The high temperature of 260 °C for a prolonged time of 1 h leads to evaporation and condensation of the solvent, benzyl ether, thus helping in the decomposition of highly stable precursors such as metal acetylacetonates. At high temperatures, the rate of the evaporation/condensation is also higher, leading to the formation of small-sized nanoparticles. After the decomposition of such metal precursors, the Triton-X100 surfactant reduces metal ions to form neutral atoms, thus initiating the nucleation of CoFe_2_O_4_ particles. In this work, nitrogen was used as an inert gas for the synthesis of CFNPs, since nitrogen is less dense than other gases such as argon, to aid in the removal of oxygen from the reaction atmosphere. After the experiments reached 100 °C, the nitrogen atmosphere was removed and the temperature increased to 260 °C. At such high temperatures, oxygen solubility in solution decreases and is highly controlled, which allows the formation of small-sized CoFe_2_O_4_ nanoparticles. The physical and magnetic structures of CFNPs in this partially controlled oxidation environment are consistent with the particles synthesized in the absence of oxygen. However, the nanoparticles synthesized in the complete absence of oxygen experienced uncontrolled aggregation and destabilization after exposure to oxygen atmosphere. Therefore, the CFNPs used in these studies were synthesized in a partially controlled oxidative environment which led to the formation of the required oxide phase, assuring the prevention of further oxidation after the synthesis. Correspondingly, there are previous reports which clearly indicate that residual oxygen plays an important role in the nucleation and controlled growth of NPs during the reaction [38].

In the absence of residual oxygen, the CFNP size increased tremendously. This may be due to fewer number of nuclei formed under the inert atmosphere, thus leading to larger sized nanoparticles. In the residual oxygen case, the number of nuclei formation increased and further growth occurred under controlled conditions, producing a smaller but more uniform particle size. Furthermore, there was a very high reproducibility in the synthesis of such partially oxygen-exposed CFNPs.

**Scheme 1:**
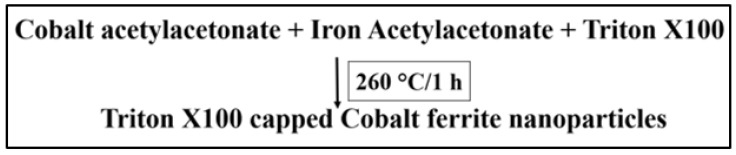
Reaction scheme.

The reaction Scheme 1 is as follows:

### 2.2. Structural Characterization

**Figure 1:**
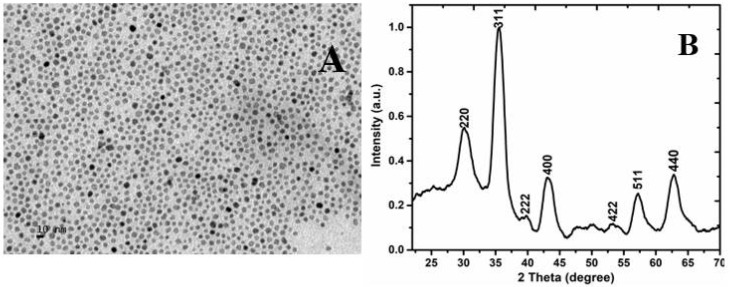
Structural characterization of cobalt ferrite nanoparticles (CFNPs). (**A**) TEM showing size of particles (**B**) XRD showing diffraction planes (220), (311), (222), (400), (422), (511) and (440) confirming CoFe_2_O_4_ presence.

Transmission electron microscope (TEM) imaging (Figure 1A) shows that the CFNPs are in the range of 5–10 nm in diameter and are spherical in shape. XRD study (Figure 1B) of the CFNPs show diffraction planes (220), (311), (222), (400), (422), (511) and (440), thus confirming that the samples possess a cubic spinel structure. The space group of CFNPs is Fd3m and corresponds to the standard JCPDS data, 22-1086. The most important observation is that the peaks are very sharp and narrow due to the very small size of the particles.

**Figure 2:**
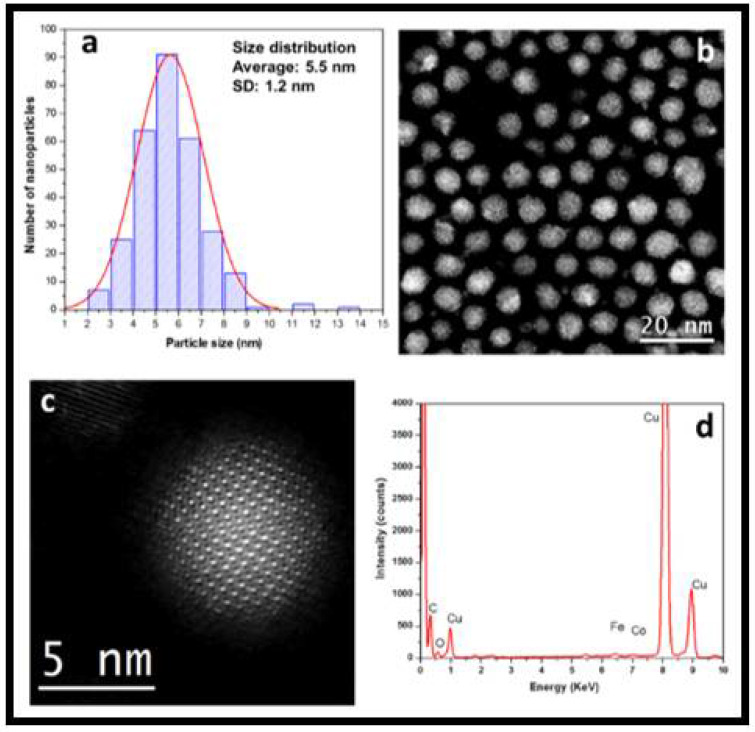
(**a**) Particle size distribution of CoFe_2_O_4_ TX100 0.1 M, (**b**) Low-magnification Cs-corrected scanning transmission electron microscopy–high-angle annular dark-field imaging (STEM–HAADF) image of CoFe_2_O_4_ TX100 0.1 M showing several NPs and a very narrow distribution, (**c**) Cs-corrected STEM–HAADF image of CoFe_2_O_4_ TX100 0.1 M showing very high resolution, the morphology corresponds to spherical NP, (**d**) EDS profiles of CoFe_2_O_4_ TX100 0.1 M NPs, the spectrum shows the elemental presence of Co, Fe, O corresponding to the NPs while C and Cu corresponds to the STEM grid.

The spherical aberration-corrected (Cs-corrected) scanning transmission electron microscopy–high-angle annular dark-field imaging (STEM–HAADF) technique was used for CFNPS and the statistical studies were also performed on several NPs. Our analysis reveals a very narrow particle size range, between 2 and 10 nm, with an average of 5.5 ± 1.2 nm, as can be observed in Figure 2a. Figure 2b revealed a very good NP Gaussian distribution, homogeneous sizing, and spherical shapes, which demonstrate good control of the synthesis method. Remarkably, we confirmed the absence of smaller or larger NPs, which can be attributed to both control of the synthesis, and the reagent and precursors chosen for synthesis. The chemical composition of the NPs was performed by spherical aberration corrected (Cs-corrected) STEM imaging. As we can observe in Figure 2c, the CFNPs are formed with a spherical shape; the size of this NP corresponds to 5 nm approximately. The crystalline structure of this NP can be described as a multi-twinned NP due to the presence of atomic columns and defects in the nanostructure. The EDS spectrum (Figure 2d) was performed on the NP shown in Figure 2c, displaying the elemental presence of Co, Fe, and O corresponding to the NP, while C and Cu corresponds to the copper grid.

### 2.3. Raman Spectroscopy

**Figure 3:**
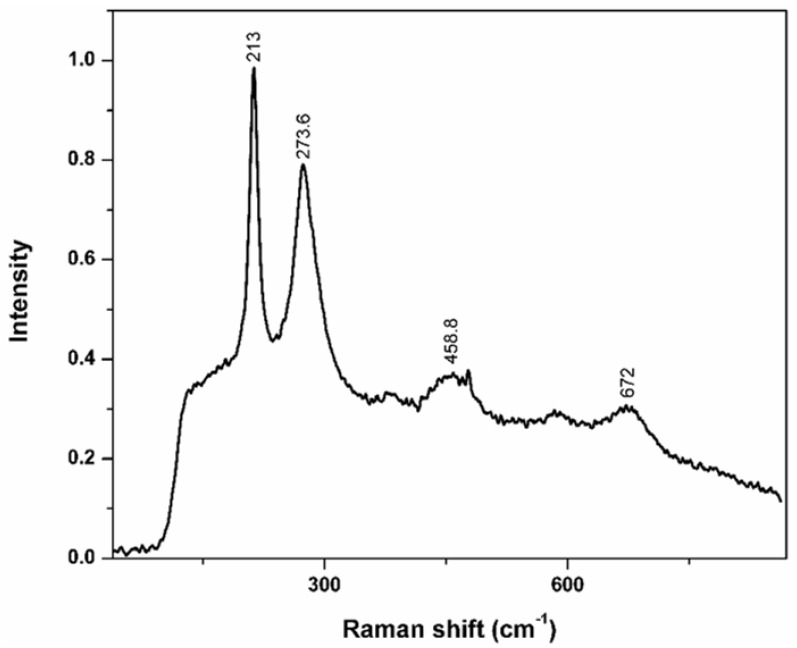
Raman spectroscopy of CFNPs, showing Raman modes at 213 cm^−1^ and 458 cm^−1^ for T_2g_, 273.6 cm^−1^ for E_g_, and 672 cm^−1^ for A_1g_.

Raman spectroscopy of the CFNPs predicts optical phonon distribution, which comprises of A_1g_, E_g_ and T_2g_, which are Raman active modes. Specifically, A_1g_ corresponds to the stretching of the oxygen anion symmetrically, E_g_ corresponds to the bending of the oxygen anion symmetrically, while T_2g_ is associated with the stretching of the oxygen anion asymmetrically with respect to the tetrahedral as well as the octahedral cations. Raman modes at 213 cm^−1^ and 458 cm^−1^ were assigned to T2g, while 273.6 cm^−1^ corresponds to E_g_, and 672 cm^−1^ is assigned to A_1g_. A_1g_ is related to the stretching vibrations of the Co-O bond in the tetrahedral site. The lower T_2g_ and E_g_ frequency modes demonstrate the vibration of the inverse spinel structure of cobalt ferrite. (Figure 3) [39].

### 2.4. Magnetic Characterization

**Figure 4:**
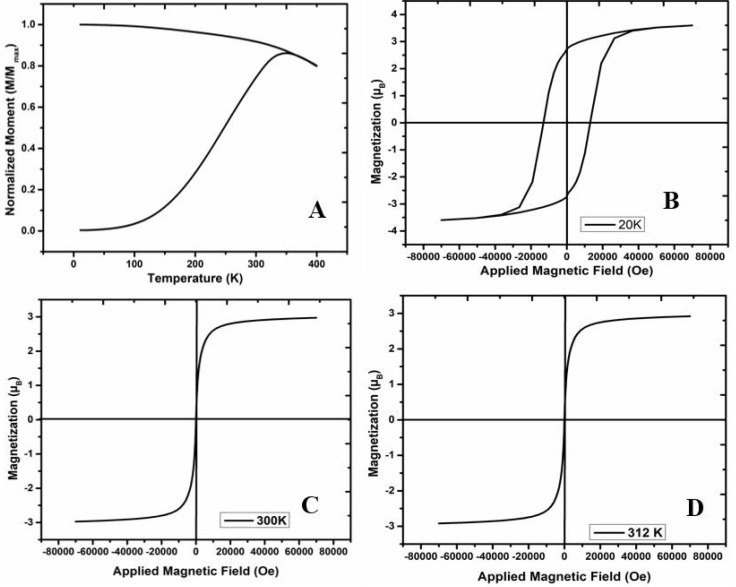
(**A**) Field cooling and zero-field cooling curves for CFNPs. Changes in magnetization as a function of applied magnetic field at different temperatures (**B**) 20 K, (**C**) 300 K and (**D**) 312 K.

Figure 4A shows the zero-field cooling (ZFC)–field cooling (FC) curves of CFNP samples measured from 10 K to 400 K. The blocking temperature is the temperature where ZFC and FC curves join together, T_B_, above which the hysteresis coercivity reduces to zero and superparamagnetic behavior begins [40]. For monodispersed non-interacting samples, this temperature region evidences a transition between irreversible and reversible regimes. CFNPs showed a T_B_ = 360 K denoted by the inflection point of the rapid magnetization rise in the FC curve up to its thermodynamic equilibrium value (when the thermal energy *k*_B_*T*_B_ is nearly proportional to the anisotropy energy barrier *KV* of the particles) [41]. Higher T_B_ indicates higher magnetocrystalline anisotropy (*K*). FC curve is flatter at lower temperatures, as depicted in Figure 4A, suggesting the existence of strong dipolar interactions among the particles [42,43,44,45], which can cause the ferrite nanoparticles to have higher anisotropy values and may increase their blocking temperatures [46].

Figure 4B–D show the hysteresis loops for CFNPs at 300 K and 312 K. In both cases, CFNPs presented similar behaviors when dispersed in Triton X (higher saturation values). The effective moment values indicated the contribution of two magnetic sublattices coupled antiferromagnetically, as expected for cobalt ferrites with two different Co(II) and Fe(III) sites. Moreover, CFNPs displayed magnetic moments reported in emu/g for Co_0.37_Fe_2.63_O_4_ and Co_0.19_Fe_2.81_O_4_ ferrites [47]. No evident traces of FeO or CoO were detected, as no exchange bias was noticed in Figure 4C,D. CFNPs displayed the higher coercive field (H_C_) at 20 K, exhibiting a value around 1.349 T (Figure 4B), thus confirming the presence of highly crystalline Co_x_Fe_3-x_O_4-δ_ nanoparticles prepared by thermal decomposition [48,49,50]. The reduced remnant magnetization (M_R_/M_S_) of CFNP is 0.86, a signature value for a system with cubic anisotropy [51].

### 2.5. Specific Absorption Rate (SAR) of CFNPs

**Figure 5:**
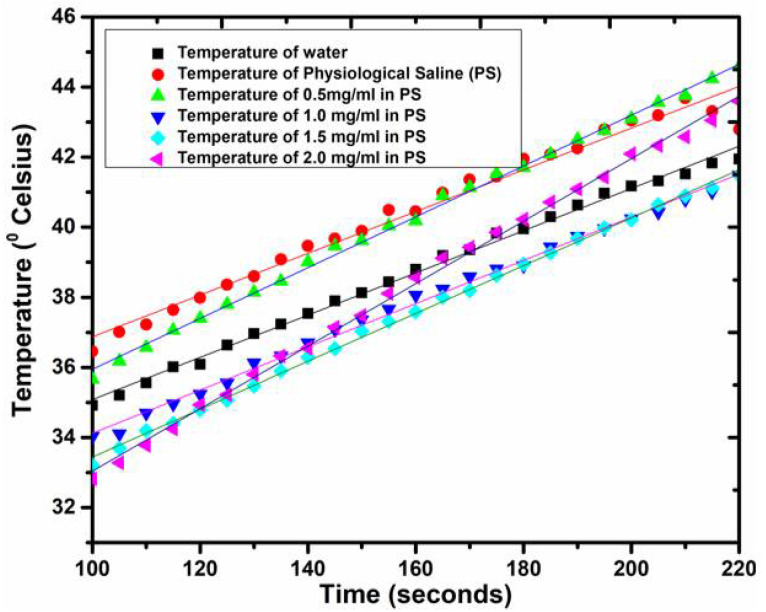
Temperature versus microwave (2.45 GHz) radiation time for water, physiological saline and different concentrations of cobalt ferrite nanoparticles in physiological saline.

Hyperthermia is a treatment strategy that involves the exposure of magnetic nanoparticles to an alternating magnetic field to increase temperature and induce apoptosis in cells or tissues. In this work, microwaves were used to irradiate CFNPs with a microcoaxial double-slot antenna as an applicator, which was placed inside the tube containing the nanoparticles in physiological saline. In this case, 2.45 GHz was used as an Industrial, Scientific and Medical (ISM)-approved frequency for localized hyperthermia induction. Figure 5 depicts the temperature profiles in correspondence to the microwave irradiation time for water and different concentrations of cobalt ferrite nanoparticles in physiological saline. The microwave absorption rate of physiological saline (3.3 × 10^−3^) is higher compared to water (1 × 10^−3^) [52]. Upon the absorption of microwaves (2.45 GHz), the rotational motion of the electric dipole of water molecules gets excited and the average phase of this motion is delayed compared to the microwave electric field. The microwave energy leads to an increase in kinetic as well as intermolecular energies of water. The kinetic energy is responsible to increase the temperature of water, while the intermolecular energy leads to internal rearrangements in the water molecules. Physiological saline suspension (diluted salt solution) is heated drastically and rapidly compared to water since salt ions tend to heat up faster, specifically Cl^−^ ions. The chloride ions in the presence of a microwave electric field tend to undergo motion and the microwave energy transfer leads to higher collision between salt ions and water molecules [53].

The specific heat capacity of 0.13 molal of physiological saline suspension is 4184 J/gram K [54]. In physiological saline, SAR was calculated as 68.28 W/g. Heat generation is possible when magnetic nanoparticles dispersed in physiological saline leads to the excitation of unpaired electron of cobalt and Fe ions to a higher-energy state when microwaves are absorbed [54]. When the excited electron alters its spin direction and returns back to its ground state, then it emits phonons. The microwave fields and the magnetic dipoles are coupled to convert the energy of radiation to heat [55]. When CFNPs present in the saline suspension are exposed to microwave radiation, then the collision between nanoparticles, salt ions and water molecules tend to increase thus leading to higher heat generation.

One of the parameters to determine the efficacy of hyperthermia is the specific absorption rate, (SAR). As mentioned earlier, SAR is defined as the amount of energy absorbed per unit mass and can be determined from the bioheating equation which, under certain experimental conditions, can be determined by the relation between the increase in temperature and time, multiplied by the specific heat capacity. This relationship of changed ratios is represented by the slope of the equation described by each of the lines in Figure 5. Considering 0.5 mg/mL as the base concentration at which all samples possess same specific heat capacity, the percentage increase in SAR for the 1.0 mg/mL, 1.5 mg/mL and 2.0 mg/mL concentrations are 3.5%, 10.7% and 30.6% respectively. As we can see, there is a non-linear increase in SAR at different concentrations. Considering that the biological effects in tissues depend on the increase in temperature and that they are also correlated with the Arrhenius equation, the increases that we obtained are significant. The Arrhenius relationship has been used to define the temperature dependence on the rate of cell killing, which can then be used as a method for thermal dosimetry in which a change in temperature of 1 °C will double the rate of cell killing, above which is known as the “break point”. Thus, the temperature increases for the different concentrations obtained are significant in relation to the biological effects.

### 2.6. In Vitro Hyperthermia Studies on TNBC Cell Lines

**Figure 6:**
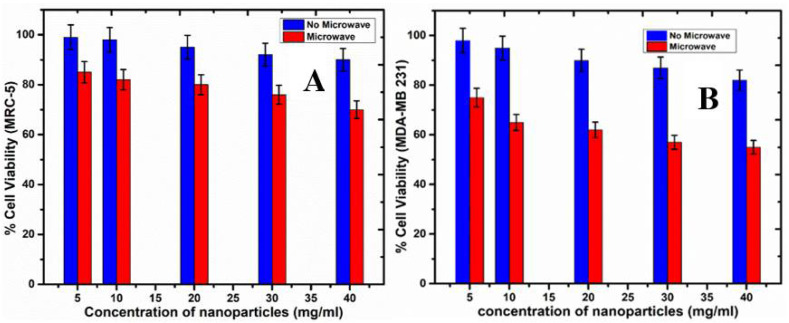
Cell viability of (**A**) MRC-5 and (**B**) MDA-MB-231 as a function of CFNP concentration with and without microwave (MW) irradiation.

Thermal exposition of the cells through microwave irradiation of the CFNPs lead to higher cytotoxicity and mortality of cells compared to the absence of microwave irradiation. In the absence of microwaves, the inherent cytotoxicity of CFNPs was less in both types of cell lines, but as soon as the cells were exposed to microwave irradiation, the viability decreased tremendously. The microwave-exposed cells also showed increasing cell mortality with increasing concentrations of CFNPs. In the presence of alternating electromagnetic radiation, the magnetic nanoparticles lead to localized hyperthermia, thus causing the degradation of the cell organelles and the fragmentation of their DNA. In Figure 6, the cell viability results of both MRC-5 and MDA-MB-231 cell lines as a function of CFNP concentrations with and without microwave irradiation are shown. Our research group found a therapeutic window of CFNPs from 5 mg/mL to 40 mg/mL in which the cell viability of MDA-MB-231 decreases to 55% in contrast to the cell viability of MRC-5 cells, showing 70% on exposure to microwave irradiation at the same CFNP concentration of 40 mg/mL. Hyperthermia can be induced in cells above 43 °C. Hence, in this study, localized hyperthermia is induced in cells using microwave irradiation, maintaining the exposure time for 50 s. There is a relationship between the microwave power output and the temperature rise in the tube containing physiological saline with cells and nanoparticles. The power output was adjusted in such a way that the temperature was maintained at 50 °C for 50 s. This optimized power output was relevant to kill cells in the presence of nanoparticles with a lower exposure time. CFNPs absorb microwaves that induce hyperthermia in the cells, thus leading to their death. In the control cell studies, the cell viability of MRC-5 upon exposure to microwaves for 50 s, but without nanoparticles, was 98%, while that of MDA-MB-231 was 94%. Cancer cells are found to be more vulnerable upon exposure to microwaves compared to the normal ones. Furthermore, when both MDA-MB 231 (triple negative breast cancer cell lines) and MRC-5 (fibroblast normal cell lines) are exposed to the same increasing concentration of the CFNPs, cancer cells are found to have more mortality compared to the normal ones upon exposure to microwaves which can be corroborated from Figure 6. The reason for this vulnerability is still not known [55].

## 3. Materials and Methods

### 3.1. Synthesis of Triton X-100 Coated CoFe_2_O_4_ Nanoparticles

The synthesis of cobalt-doped Fe_3_O_4_ magnetite NPs was carried out by thermal decomposition (TD), which consists of the precursor reaction at 260 °C to produce NPs. For the TD process, the following chemicals were mixed in 20 mL benzyl ether (Sigma Aldrich, St. Louis, MO, USA, 98%) solution: iron (III) acetyl acetonate (1 mmol, Sigma-Aldrich 97%), cobalt acetyl acetonate (0.5 mmol Sigma Aldrich 97%) and an organic surfactant of 0.1 M Triton X-100. Before heating, the solution was exposed to a nitrogen atmosphere (N_2_) to remove oxygen and other gases from the solution. Then, the temperature was increased to 100 °C and the N_2_ gas exposure was discontinued. The temperature was then increased to 260 °C and maintained for 60 min. This leads to the formation of monodisperse CoFe_2_O_4_ nanoparticles, which possess a characteristic dark brown color. The solution was left at room temperature for 24 h and subsequently rinsed and centrifuged for 5 cycles at 8000 RPM. In the first three cycles, 5 mL of 98% methanol was used, while in the last two cycles, 5 mL of double-distilled water was used for washing the magnetic nanoparticle solution. After centrifugation and washing, the precipitate was added into 15 mL of double-distilled water.

### 3.2. Characterization of CFNPs

The X-ray diffraction (XRD) pattern of the cobalt ferrite powder was obtained through XRD measurement (X’Pert PRO spectrometer–PANalytical, Malvern, UK) using Cu Kα radiation (λ = 0.154 nm). Raman analysis was performed on CFNPs using Micro-Raman LabRAM Jobin Yvon scientific X’plora with a 638-nm wavelength red emission laser and a 50× objective lens. The morphology and confirmation of cobalt ferrite nanoparticle formation was characterized using transmission electron microscopy (TEM JEM-ARM200F, Jeol, Tokyo, Japan) along with other related techniques such as HR-TEM (High Resolution Transmission Electron Microscopy), HAADF-STEM (High-Angle Annular Dark-Field Scanning Transmission Electron Microscopy), and EDS (Energy Dispersive X-Ray Spectrometry) (Oxford XMax 80). Magnetic measurements were performed on a superconducting quantum interference device (SQUID), (Quantum Design, MPMS3). Zero-field cooling (ZFC), field cooling (FC) and hysteresis curves were measured on the MPMS3 apparatus. ZFC/FC curves were collected by first cooling down the samples from 400 K to 10 K under a zero magnetic field, then the magnetic moment was measured as the temperature was increased from 10 K to 400 K under a 100-Oe applied magnetic field. Hysteresis curves were taken from −7 to 7 T in a magnetic field at 20 K, 300 K and 312 K.

### 3.3. In-Vitro Hyperthermia Effects of Cobalt Ferrite Nanoparticles

A pre-assembled homemade setup was used to perform the microwave (MW)-based hyperthermia studies at 2.45 GHz. The homemade MW experimental setup consisted of the following components: a frequency generator (SML 03, Rohde & Schwarz, Skarpnäck, Sweden), MW signal amplifier (1164-BBM3Q6AHM, Empower), output power monitor (DC7154 M, Amplifier Research), incident and reflected power monitor (PM2002, Amplifier Research), standing wave ratio controller (SWR coaxial stub tuner 1878C, Maury Microwave Corp) and network analyzer (E5071B, Agilent Technologies). The temperature change in the sample was measured using non electromagnetic interfering optical fiber sensors (M3300, Luxtron). The output power of the generator was −13.7 dBm, which, when passing through the amplifier module, reached a power output of 3 W (34.77 dBm). The bidirectional coupler consisted of two power sensors that allow for the continuous measuring of both incident and reflected power. The impedance coupler was used to adjust the impedance of the antenna to 50 ohms since the impedance of the antenna could vary depending on the medium in which it radiates and is immersed. The thermal container allowed us to avoid the heat exchange of the sample with the environment during the application of electromagnetic energy, which lasted three minutes. The antenna was covered with Teflon tape to isolate the antenna, which was made of copper, with the material to be radiated. Optical fiber 1 was placed without a capillary tube to have a distance of 0 mm from the antenna and optical fibers 2 and 3 were placed inside capillary tubes to maintain a separation of 1 mm and 2 mm, respectively, from the antenna, as shown in Figure S1. The tip of the optical fibers was placed in the center of the antenna slot, which was the place where the greatest amount of radiated energy is concentrated. Teflon tape was used to fix the array of optical fibers to the antenna and to fix the same height of the antenna within the samples, as indicated in Figure S1. The computer allowed us to store information on the temperature of the optical fibers.

To produce the microwave radiation, a double-slot microcoaxial antenna was placed in a physiological saline suspension containing different concentrations of CFNPs in the order of 0.5–2 mg/mL, while the temperature increment was measured with the help of a non-interfering fiber optic probe. SWR for all the experiments was measured and adjusted to near 1.0, which guarantee electromagnetic energy transfer to the samples from the radiation equipment. For calibration, microwave exposure was performed for 300 s at 6 W while the temperature increment was measured. Later, in vitro hyperthermia studies were performed on both normal cells (MRC-5) and cancer cells (MDA-MB-231).

MRC-5 normal fibroblast cell lines from lung tissue were cultured in Dulbecco’s Modified Eagle Medium (DMEM) culture medium (10%), fetal bovine serum (10%), L-glutamine, and amino acids at 37 °C with a 5% CO_2_ atmosphere. MDA-MB-231 TNBC cell lines were cultured in RPMI1640 culture medium, containing the antibiotics streptomycin and penicillin (100 mg/mL) and 10% fetal bovine serum without amino acids. Cytotoxicity measurements were performed on the CFNPs in the presence and absence of microwave-based hyperthermia.

To evaluate cell viability, both MRC-5 and MDA-MB-231 were seeded at a density of 5 × 10^4^ cells cultured into a 24-well plate and incubated in 5% CO2 atmosphere at 37 °C for 24 h. After 24 h, various concentrations of cobalt ferrite nanoparticles in the range of 50–250 mg/mL were added in the respective wells. Two sets of the same concentration of cobalt ferrite nanoparticles were prepared. The cells were incubated with the nanoparticles for 2 h and then one set was used for microwave-based hyperthermia, while the other set was kept as control without any exposure to microwave radiation. The microwave-exposed cells with increasing concentrations of cobalt ferrite nanoparticles showed increased cell mortality after exposure to microwave irradiation of 2.4 GHz for 50 s at 50 °C. This experiment of two sets was performed in triplicates. After the hyperthermia treatment, the cells were then again placed back in the well with fresh medium added and incubated in the 24-well plate in 5% CO2 atmosphere for 24 h at 37 °C. After 24 h, 10 μL of MTT reagent (3-(4,5-dimethylthiazol-2-yl)-2,5-diphenyl tetrazolium bromide) (5 mg/mL) was pipetted in each well containing cells and incubated for 3 h. After the incubation period, the absorbance was recorded at 490 nm using a microplate reader in triplicate and the average values were considered.

## 4. Conclusions

In this work, thermal decomposition synthesis of CoFe_2_O_4_ nanoparticles was successfully performed in a controlled manner to produce homogenous, uniformly sized nanoparticles. At the higher temperatures employed in this study, the oxygen solubility was lower and hence formed a smaller oxide layer during the synthesis reaction, allowing for better and more reliable particle formation. The purified nanoparticles redispersed in physiological saline were then used to perform in vitro hyperthermia studies on TNBC cell lines and fibroblast cells. Our research group achieved an adequate design, morphological structure, and composition of CFNPs for hyperthermia treatment. In addition, we determined a therapeutic window from 5 mg/mL to 40 mg/mL for this configuration of NPs; therefore, these NPs are good candidates for the continuation of drug nanofunctionalization in the future.

## Supplementary Materials

The following are available online. Figure S1: Microwave based-Hyperthermia set-up.
