# Supplementary material for: Synthesis, Characterization and Magnetic Hyperthermia of Monodispersed Cobalt Ferrite Nanoparticles for Cancer Therapeutics
Source: Molecules. 2020 Sep 27;25(19):4428. doi: 10.3390/molecules25194428 (PMC7583941; doi:10.3390/molecules25194428)
Supplement: Supplementary file 1 [file molecules-25-04428-s001.pdf]

molecules

Article

Synthesis, Characterization and Magnetic Hyperthermia of Monodispersed Cobalt Ferrite Nanoparticles for Cancer Therapeutics

Mauricio A. Medina [1,*], Goldie Oza [2,*], A. Ángeles-Pascual [3], Marlene González M. [4], R. Antaño-López [2], A.Vera [5], L. Leija [5], Edilso Reguera [6], L. G. Arriaga [2], José Manuel Hernández Hernández [7] and José Tapia Ramírez [8,*]

1 Program on Nanoscience and Nanotechnology, CINVESTAV-IPN, Avenida IPN 2508, Gustavo A. Madero, San Pedro Zacatenco, 07360, México city, Mexico
2 Centro de Investigación y Desarrollo Tecnológico en Electroquímica (CIDETEQ), Parque Tecnológico Querétaro s/n, Sanfandila, Pedro Escobedo, Querétaro C.P. 76703, Mexico; rantano@cideteq.mx (R.A.L); larriaga@cideteq.mx (L.G.A.)
3 Laboratorio Avanzado de Nanoscopía Electrónica-LANE, CINVESTAV-IPN, Avenida IPN 2508, Gustavo A. Madero, San Pedro Zacatenco, 07360, México city, Mexico; aangelesp@cinvestav.mx
4 CONACyT-Instituto Politécnico Nacional, Centro de Investigación en Ciencia Aplicada y tecnología Avanzada, U. Legaria, Ciudad de México 11500, Mexico; maglerne@gmail.com
5 Bioelectronics Section, Department of Electrical Engineering, CINVESTAV-IPN, Avenida IPN 2508, Gustavo A. Madero, San Pedro Zacatenco, 07360, México city, Mexico ; arvera@cinvestav.mx (A.V.); lleija@cinvestav.mx (L.L.)
6 Instituto Politécnico Nacional, Centro de Investigación en Ciencia Aplicada y tecnología Avanzada, U. Legaria, Ciudad de México 11500, Mexico; edilso.reguera@gmail.com
7 Department of Cell Biology, CINVESTAV-IPN, Avenida IPN 2508, Gustavo A. Madero, San Pedro Zacatenco, 07360, México city, Mexico; manolo@cell.cinvestav.mx
8 Department of Genetics and Molecular Biology, CINVESTAV-IPN, Avenida IPN 2508, Gustavo A. Madero, San Pedro Zacatenco, 07360, México city, Mexico.
* Correspondence: mauricio.medina@cinvestav.mx (M.A.M.); goza@cideteq.mx (G.O.); jtapia@cinvestav.mx (J.T.R.)

Received: 6 August 2020; Accepted: 18 September 2020; Published: 27 September 2020

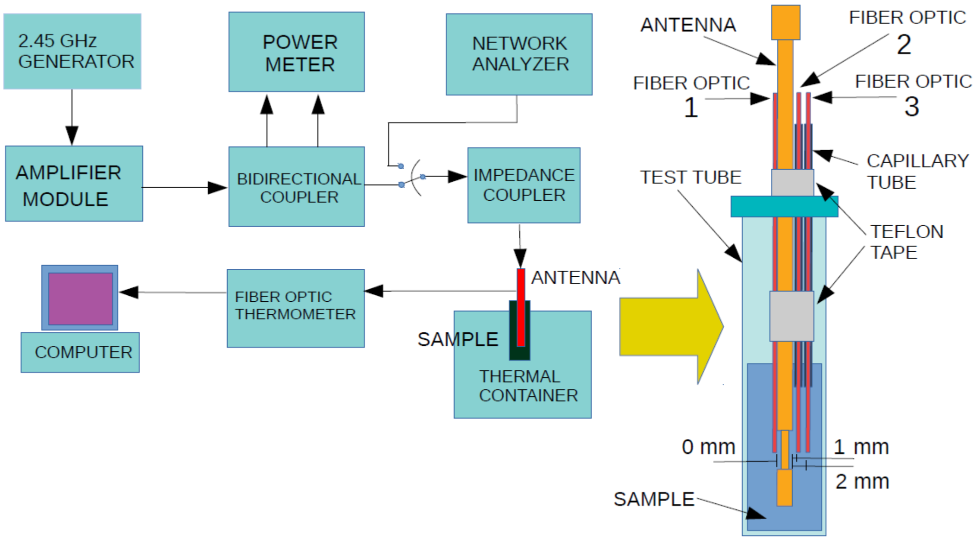

Figure S1. Microwave based-Hyperthermia set-up.
